# First report of *Setaria tundra* in roe deer (*Capreolus capreolus*) from the Iberian Peninsula inferred from molecular data: epidemiological implications

**DOI:** 10.1186/s13071-016-1793-x

**Published:** 2016-09-29

**Authors:** Samer Angelone-Alasaad, Michael J. Jowers, Rosario Panadero, Ana Pérez-Creo, Gerardo Pajares, Pablo Díez-Baños, Ramón C. Soriguer, Patrocinio Morrondo

**Affiliations:** 1 Estación Biológica de Doñana, Consejo Superior de Investigaciones Científicas (CSIC), Sevilla, Spain; 2 Institute of Evolutionary Biology and Environmental Studies (IEU), University of Zurich, Zurich, Switzerland; 3 CIBIO/InBIO (Centro de Investigação em Biodiversidade e Recursos Genéticos), Universidade do Porto, Campus Agrario De Vairão, 4485-661 Vairão, Portugal; 4 National Institute of Ecology, 1210, Geumgang-ro, Maseo-myeon, Seocheon-gun, Chungcheongnam-do 33657 Korea; 5 INVESAGA Group, Departamento de Patología Animal. Facultad de Veterinaria, Universidad de Santiago de Compostela, 27071, Lugo, Spain

**Keywords:** Filarioid, Mitochondrial cytochrome *c* oxidase subunit 1 (*cox*1), Phylogenetics, Epidemiology, Climate change, Bayesian inference analysis

## Abstract

**Background:**

Filarioid nematode parasites are major health hazards with important medical, veterinary and economic implications. Recently, they have been considered as indicators of climate change.

**Findings:**

In this paper, we report the first record of *Setaria tundra* in roe deer from the Iberian Peninsula. Adult *S. tundra* were collected from the peritoneal cavity during the post-mortem examination of a 2 year-old male roe deer, which belonged to a private fenced estate in La Alcarria (Guadalajara, Spain). Since 2012, the area has suffered a high roe deer decline rate (75 %), for unknown reasons. Aiming to support the morphological identification and to determine the phylogenetic position of *S. tundra* recovered from the roe deer, a fragment of the mitochondrial cytochrome *c* oxidase subunit 1 (*cox*1) gene from the two morphologically identified parasites was amplified, sequenced and compared with corresponding sequences of other filarioid nematode species. Phylogenetic analyses revealed that the isolate of *S. tundra* recovered was basal to all other formely reported *Setaria tundra* sequences. The presence of all other haplotypes in Northern Europe may be indicative of a South to North outbreak in Europe.

**Conclusions:**

This is the first report of *S. tundra* in roe deer from the Iberian Peninsula, with interesting phylogenetic results, which may have further implications in the epidemiological and genetic studies of these filarioid parasites. More studies are needed to explore the reasons and dynamics behind the rapid host/geographic expansion of the filarioid parasites in Europe.

## Background

Filarioid nematode parasites are major health hazards with significant medical, veterinary and economic implications, with millions of people and animals globally affected [1]. Haematophagous arthropods are the vectors of these parasites [2], which have recently been considered as indicators of climate change [3, 4]. Filarioid parasites are difficult to control due to vectors’ mobility and/or the risk of resistance to drugs [5].

The phylogenetic relationships of filarioid parasites have been assessed on morphological characters [6, 7]. Nevertheless, molecular analyses are needed to confirm their morphological descriptions or taxonomic position and classification, and to improve our understanding of the species epidemiology [5, 8].

In Europe, *Setaria tundra* has been reported from Germany [9, 10], Bulgaria [11], Sweden [12], Norway [13], the Baikal area [14], Italy [15], Poland [16] and Finland [17]. *Setaria tundra* is considered as a common parasite in the northern hemisphere, especially in Finland, where mass infection has occurred [17]. Here there seems to be a correlation of infection to wetland areas, where *S. tundra* vectors (mosquitoes) are at their optimal microclimate condition and reindeer herds are present [18], with 30–40 % of all *S. tundra* outbreaks in Finland.

The aims of the present study were to: (i) Report for the first time *S. tundra* in the roe deer *Capreolus capreolus* from the Iberian Peninsula, and (ii) examine the phylogenetic position of the newly-found *S. tundra*, based on sequences of the mitochondrial cytochrome *c* oxidase subunit 1 (*cox*1) gene.

## Methods

### Case report, sample collection and morphological examination

During the last 3 years, a private fenced estate in La Alcarria (Guadalajara, Spain) has suffered from a decline of 75 % of roe deer population for unknown reasons. Moreover, lower body weight and reduction of trophy size in males have been noted. Roe deer in this fenced estate are native animals (the density before decline was *c.*30 animals per 100 ha). In the study area, there are no domestic animals. The sympatric wild animals are the wild boar *Sus scrofa*, the red fox *Vulpes vulpes* and the European badger *Meles meles*, together with different bird species, which interact with roe deer, namely the golden eagle *Aquila chrysaetos*, the griffon vulture *Gyps fulvus* and the cinereous vulture *Aegypius monachus*.

**Fig. 1:**
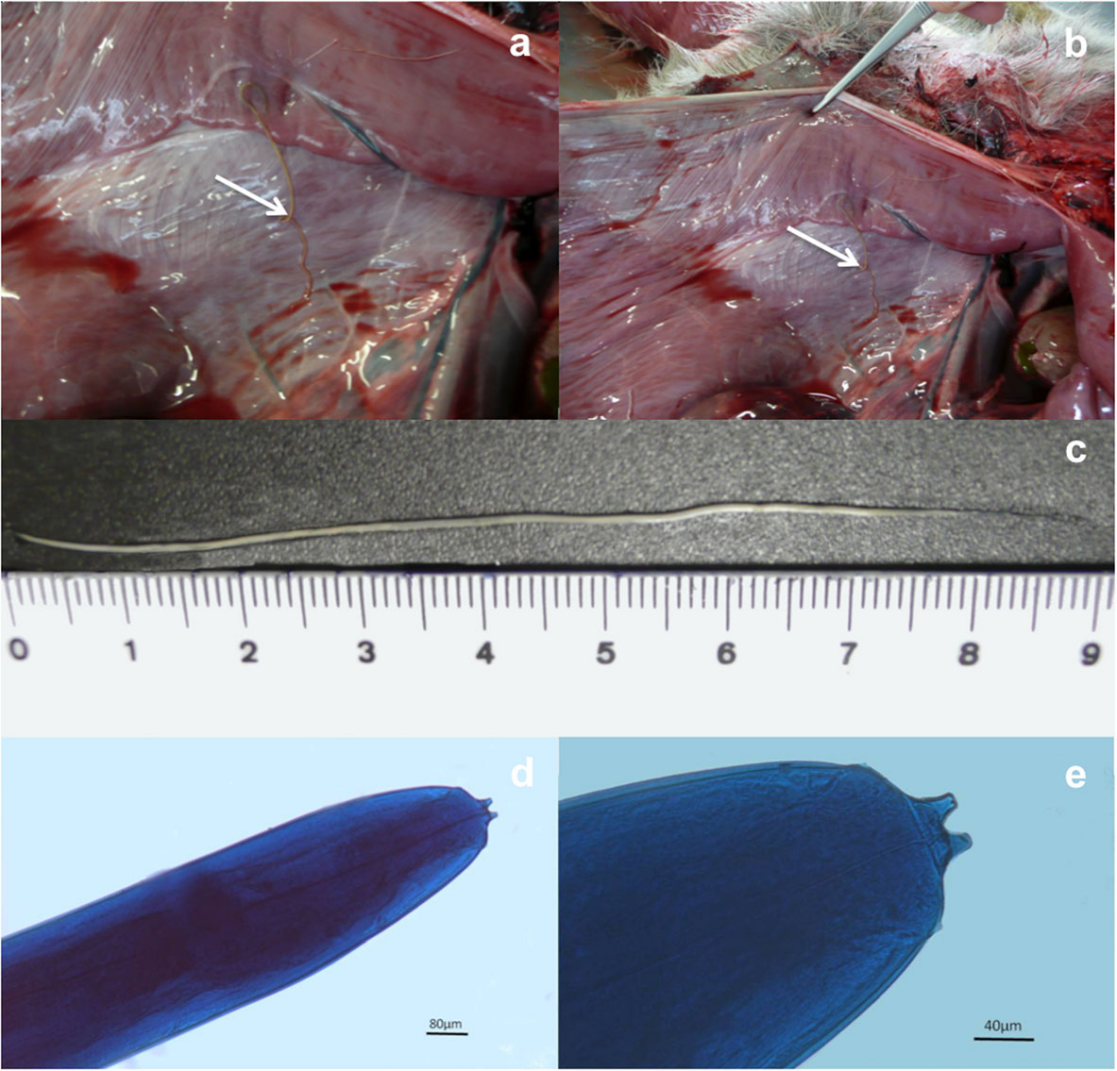
Images showing **a**, **b** necropsy of the peritoneal cavity of the roe deer with adult *Setaria tundra*, **c** the recovered parasites, and photomicrographs of *S. tundra* (**d**, **e**)

To identify the reason behind the high population decline, the owner sent on the 5th of April 2016, the carcass of a 2 year-old male roe deer to the INVESAGA laboratory at the Faculty of Veterinary Medicine in Lugo for post-mortem examination. The animal was apparently in poor body condition. During necropsy, two adult females of *Setaria tundra* were collected from the peritoneal cavity (Fig. 1). Nematodes were preserved at room temperature in 70 % ethanol and then stained with 0.01 % cotton blue in lactophenol before morphological identification, and later DNA extraction. *Setaria* specimens were identified as *S. tundra* based on morphological characteristics described in [19] (Fig. 1).

### DNA extraction

Genomic DNA was extracted from each of the two *S. tundra* specimens using DNeasy Blood & Tissue Kit (Quigen, Hilden, Germany). DNA extractions were carried out in a sterilized laboratory dedicated exclusively for low DNA concentration samples. Two blanks (reagents only) were included in each extraction to monitor for contamination.

### PCR and sequencing of the mitochondrial *cox*1 gene

The methods of Casiraghi et al. [20] for PCR amplification of the partial *cox*1 gene, were followed. The 30-μl PCR mixture contained 2 μl of template DNA, 0.25 μM of the primers cox1intF (5′-TGA TTG GTG GTT TTG GTA A-3′) and cox1intR (5′-ATA AGT ACG AGT ATC AAT ATC-3′), 0.12 mM of each dNTP, 3 μl of PCR buffer (Bioline, Sydney, Australia), 1.5 mM MgCl_2_, 0.4 % BSA, 1.5 μl DMSO, and 0.2 μl (0.2 U/reaction) Taqpolymerase (Bioline). The following thermal profile for amplification in a PTC0200 thermal cycler (Bio-Rad) was used: 4 min at 94 °C (initial denaturation), followed by 30 cycles of three steps of 1 min at 94 °C (denaturation), 1 min at 52 °C (annealing), and 50 s at 72 °C (extension), before a final elongation of 5 min at 72 °C. PCR blanks (reagents only) were included.

Sequencing was carried out in both directions using the BigDye® Terminator v1.1 cycle sequencing kit (Applied Biosystems) according to the manufacturer’s instructions. Labelled fragments were resolved on an automated A3130xl genetic analyser (Applied Biosystems).

### Molecular analyses

Amplicons were sequenced on both strands, and the complementary reads were used to resolve rare, ambiguous base-calls in Sequencher v.4.9. Additionally, BLAST searches were conducted in GenBank and matches with high genetic affinity were downloaded and included in the alignment, with a cut-off point at 91 % genetic similarity. Higher genetic divergence recovered from other homologous sequences from *Setaria* spp. were downloaded and included in the alignment. The selected outgroup was *Thelazia callipaeda*. Sequences were aligned in SeaView v.4.2.11 [21] under ClustalW2 [22] default settings. The most appropriate substitution model for the Bayesian Inference (BI) analysis was determined by the Bayesian Information Criterion (BIC) in jModeltest v.2 [23]. MrBayes v.3.2.6 [24] was used with default priors and Markov chain settings, and with random starting trees. Each run consisted of four chains of 20,000,000 generations, sampled each 10,000 generations and posterior distributions of parameter estimates were visually inspected in Tracer v1.5 [25]. A plateau was reached after few generations with 25 % of the trees resulting from the analyses discarded as ‘burn-in’. Phylogenetic relationships among haplotypes were estimated using a Maximum Likelihood (ML) approach, as implemented in the software RAxML v7.0.4 [26], using the default settings. The 50 % bootstrap consensus tree was built in PAUP 4 [27]. All analyses were performed through the CIPRES platform [28].

## Results and discussion

In this paper, we report the first record of *Setaria tundra* in roe deer from the Iberian Peninsula. The parasites were identified morphologically and genetically, based on the sequences of a fragment of the mitochondrial cytochrome *c* oxidase subunit 1 (*cox*1) gene. The newly-obtained sequences were submitted to GenBank (KX599455–KX599456). The alignment length was 637 bp. GenBank blast matched *Setaria tundra*, with 99 % similarity and a highest divergence (similarity of 98 %) to the isolate 71YT MNHN (KP760209) with 10 bp substitutions. The closest match of the present isolate was to isolates 6615 (KF692104) and 5808 (KF692103) from Germany, with only two bp substitutions.

**Fig. 2:**
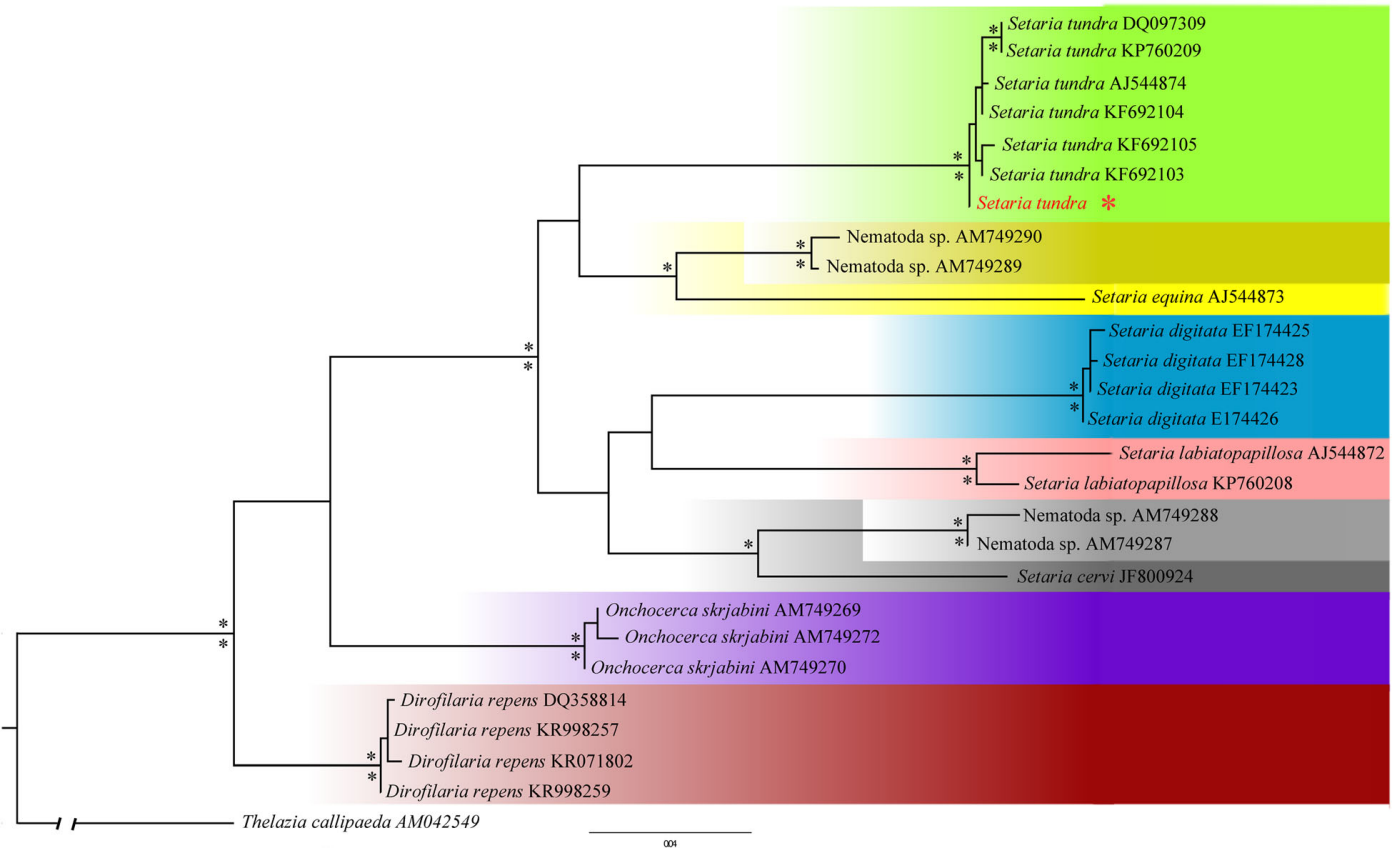
Best maximum likelihood (ML) tree for the *cox*1 (637 bp) dataset of filarioid sequences including the newly-recovered *Setaria tundra* haplotype in *red*. Asterisk (*) on and under nodes are posterior probabilities (Pp) recovered from the Bayesian analysis and bootstrap support from the ML 50 % majority-rule consensus tree (≥ 95 %), respectively. The following GenBank accession numbers recovered the same haplotypes and only one GenBank sequence per haplotype is included in the tree: *Onchocerca skrjabini* (AM749269, AM749271), *Dirofilaria repens* (AB973225, KT901783, DQ358814), *Setaria digitata* (EF174428, EF174427), *Setaria digitata* (EF174424, EF174423, GU138699), *Setaria tundra* (KF692104, KK692106), *Setaria tundra* (KM452922, AM749298, KF692103)

In the phylogenetic analysis, the best-fitting model identified was the TrN + I + G (−lnL = 2610.81847, BIC = 5602.586350). The Effective Sample Size (ESS) values for all runs were over 1,800, thus confirming good convergence mixing of all mcmc (Markov Chain Monte Carlo) runs. All analyses recovered a well-resolved monophyletic clade of *Setaria tundra* (Bayesian posterior probability (Pp) of 1; ML bootstrap 100 %) with the position of the new sequence basal to the remaining haplotypes (Fig. 2). All terminal clades recovered high support in all analyses and all species were monophyletic (Pp: 1.00).

The recovered phylogenetic tree showing two monophyletic clades of unidentified nematodes within *Setaria* may suggest paraphyly of the genus (Fig. 2), which demands further taxonomic assessment within *Setaria.* The basal positioning of *Setaria tundra* recovered from this study suggests ancestry to all known European *S. tundra* haplotypes. Thus, although ancestral, its presence may have been undetected until now by limited case studies such as this one. The presence of all other haplotypes in northern Europe may be indicative of a South to North European outbreak route rather than the opposite. Further molecular taxonomic identification is needed to assess the extent of the parasite range in the Iberian Peninsula, the host species and parasite genetic diversity. Through such sampling the ancestral and descendent populations could elucidate important information regarding intermediate populations likely to be infected by *Setaria* that are currently unrecorded.

Other studies have found *C. capreolus* to be the host for *S. tundra* in Germany [29], Bulgaria [11], Italy [15], Finland [30] and Poland [16]. Our study thus increases the host infective range from Northern to Southern Europe, as well as the parasite range. Several are the candidate insect vectors for *Setaria* spp. infections [17, 31]. Mammophilic mosquitoes, *Aedes* spp. and *Anopheles* spp., have shown to have an important role in the transmission of *S. tundra* in Finland, which suggests that this parasite is probably not vector-specific, and this consequently enhances the ability of *S. tundra* to expand geographically. Infections with *S. tundra* in reindeer herds in Finland are associated to wetlands, with fresh food pastures and drinking water, where mosquito populations are at their optimum microclimate condition to thrive [18]. The presence of *S. tundra* here seems to be related to herd migrations, with a decline in the presence of *S. tundra* in hosts or mosquitoes in the Upper Lapland [17, 32]. These data are key to assess the likely areas to conduct surveys in the Iberian Peninsula and to establish molecular screening protocols for possible vectors and hosts. The ecological and topographical conditions at the La Alcarria conform to the vectors’ optimal environmental conditions. Furthermore, the presence of permanent water and the closure of the herd prevent animals to avoid possible high-risk infectious areas by migration. The Scandinavian (1973) *S. tundra* outbreak was associated to an exceptional warm period and high numbers of possible vectors such as mosquitoes and gnats [33]. Thus, further studies are needed to assess the possible impact of climate change on transmission dynamics of *Setaria* spp. in Europe [4].

## Conclusions

This is the first report of *Setaria tundra* in roe deer from the Iberian Peninsula, with interesting phylogenetic results, which may have further implications in the epidemiological and genetic studies of filarioid nematode parasites. More studies are needed to explore the reasons and dynamics behind the rapid host/geographical expansion of filarioid parasites in Europe.
